# Dental and medical health status and oral health knowledge among visually impaired and sighted female schoolchildren in Riyadh: a comparative study

**DOI:** 10.1186/s12903-017-0446-6

**Published:** 2017-12-19

**Authors:** Salwa A. AlSadhan, Asma M. Al-Jobair, Mariam Bafaqeeh, Hanadi Abusharifa, Maram Alagla

**Affiliations:** 1 Department of Periodontics and Community Dentistry, College of Dentistry, King Saud University, P. O. Box 60169, Diriyah, Riyadh, 11545 Saudi Arabia; 2 Department of Pediatric Dentistry and Orthodontics, College of Dentistry, King Saud University, Riyadh, Saudi Arabia; 3 College of Dentistry, King Saud University, Riyadh, Saudi Arabia

**Keywords:** Visually impaired children, Oral health, Plaque scores, Gingival scores

## Abstract

**Background:**

The impact of visual impairment on oral health in the literature is inconclusive, and the available information on the medical and dental health status of visually impaired children is limited. The aim of this study was to evaluate the dental and medical health status, and to assess the oral health knowledge of visually impaired girls aged 6–12 years, and compare them to that of sighted children.

**Methods:**

This analytical cross-sectional study was carried out on 79 visually impaired and 83 age-matched sighted female primary school children. The children’s demographic data, medical history, and dental history were obtained through a validated questionnaire. The study population was examined to evaluate their dental caries status using the Decayed Missing Filled Teeth/Surface indices DMFT/DMFS/ and dmft/dmfs for permanent and primary teeth, respectively. Oral hygiene index (OHI), Plaque index (PI) and gingival index (GI) were obtained for periodontal evaluation. Pearson’s Chi-square test and t-test were used for the statistical analyses.

**Results:**

The general health for both groups was found to be good; however, 21.5% of the visually impaired children had systemic diseases compared with only 4.8% of the sighted children (*P* = 0.002). Statistically significant differences (*P* < 0.001) were found between the two groups with regards to OHI. Among the sighted children, 49.4% had good oral hygiene compared with only 22.8% of the visually impaired group. The plaque accumulation was found to be greater among the visually impaired group and gingivitis was also higher. The DMFS score was found to be higher (*P* = 0.03) among the visually impaired group.

**Conclusions:**

The visually impaired children had more medical conditions and poorer oral health status compared to their sighted peers.

## Background

Many people believe that an attractive smile and good teeth are a manifestation of good oral health. In fact, oral health contributes to self-confidence and general systemic health [1]. People acquire their oral care habits as they grow up and carry them on into adolescence. Therefore, it is important for children to practice oral hygiene at an early age and maintain it throughout life. The maintenance of oral hygiene among visually impaired children may be quite challenging, and there is utmost need for oral health supervision to control plaque [2]. The recent World Health Organization (WHO) estimate of the blind population is 285 million worldwide, and an estimated 19 million of these are children [3]. According to the WHO, blindness is defined as visual acuity of less than 3/60, or a corresponding visual field loss to less than 10°, in the better eye with the best possible correction. ‘Visual impairment’ includes both low vision and blindness. Several previous studies reported that visually impaired children had poor oral hygiene compared with their normal sighted peers [4–7]. However, one study found that visually impaired subjects demonstrated better oral hygiene practices than sighted ones [8]. Another study found no significant difference between the two groups [3]. As for dental knowledge, it was found that students with visual impairments were less knowledgeable about their oral care [9].

Dong and Dawes [10] reported that the unstimulated salivary flow rate of blind and blindfolded people was significantly reduced, highlighting the effect of blindness on the oral cavity. Another study suggested that the reason for higher rates of oral health diseases among disabled children was that oral health education is neglected while the focus is kept predominantly on managing their existing disability [1]. Conventional methods simplify oral health education for sighted children by making use of visual interaction, such as demonstrating brushing techniques using jaw models or using disclosing agents. Such techniques are considered inconvenient for visually impaired children [2, 8]. In addition, medical conditions associated with blindness might have an impact on the oral health status of visually impaired children. A study conducted on blind schoolchildren found that blindness or low vision had psychological implications including sadness, anxiety and depression [11].

Few studies have been conducted to determine the dental health status and knowledge of visually impaired children in Saudi Arabia [12–14]. To our knowledge, no previous study has been conducted among visually impaired 6–12-year-old children in Riyadh to evaluate their medical and dental conditions. Therefore, the aim of this study was to evaluate the dental and medical health status, and to assess the oral health knowledge of visually impaired 6–12-year-old children, and compare them with the status of sighted children.

## Methods

This analytical cross-sectional study commenced after granting an ethical approval from the CDRC (Ethical Committee of the College of Dentistry Research Center) at King Saud University (Reg. No. IR0099). Permission letters were obtained from the Ministry of Education and were forwarded to the school’s authorities to allow the distribution of questionnaires and examination of the children. The study was conducted between July 2014 and March 2015.

The study was carried out on 162 female primary school children aged 6–12 years old (1st–6th grade). All of the accessible visually impaired female children in the selected age group (*n* = 79) in Riyadh were included as the study group, and 83 sighted children were randomly selected to serve as the control group according to the age and school of the visually impaired group. Twenty-eight of the selected visually impaired children were studying at Al-Noor school, which is a specialized school for visually impaired children. The remaining 51 students were recruited from the four primary public schools with an inclusive education system for visually impaired children in Riyadh. These schools were distributed among the different regional areas in Riyadh and the control group was selected from these same schools.

A 16-item questionnaire was used in this study [15, 16]. It recorded the children’s demographic data, medical and dental history, oral health knowledge, sources of information about oral health, and oral hygiene habits. The questionnaire was translated from English into Arabic and was modified to suit the study objectives. The questionnaire was validated and then distributed among the 162 participating children to be answered by their parents.

The objectives of the study and its protocol were clarified in the questionnaire’s cover page. It was also explained in the cover page that the parents’ answering the questionnaire was considered consent for their children to participate in this study.

One trained examiner examined all the children. Ten percent of the children (*n* = 16) were randomly selected to be re-examined to check the intra-examiner reliability. A small portable dental chair was used for the examination of the children in their schools, using a penlight and a disposable plastic examination kit (plastic mouth mirror, explorer, cotton pliers) in addition to personal protective barriers (gloves and masks). All examination procedures were briefly explained to each child before they began. The Tell-Feel-Do principle was applied for the visually impaired children by allowing them to touch and feel the dental instruments used before the examination took place. The children’s behavior was assessed during the examination procedure. The obtained data were recorded by one recorder on a previously developed examination form [15, 16]*.* The examination form encompassed a periodontal evaluation including oral hygiene, plaque and gingival scores. In addition, assessment of the prevalence and severity of dental caries was carried using the Decayed, Missing and Filled Teeth/Surfaces (DMFT/ DMFS) indices for permanent teeth, and the decayed, missing and filled teeth/surfaces (dmft/ dmfs) indices for primary teeth, according to WHO criteria (2013) [17]. In all children, gingival status as measured by the oral hygiene index (OHI) by James et al. (1960) [18] was used to assess oral hygiene, Loe’s (1967) [19] plaque index (PI) was used to record the plaque deposition, and the gingival index (GI) described by Nanda (1990) [20] was used to evaluate the gingival health condition. These indices are popular and frequently used in studies conducted in Saudi Arabia. They are also often cited in other similar studies. The Frankl Scale (1962) [21] was used to assess the children’s behavior; the child’s reaction to dental treatment was rated on a four-point scale ranging from definitely negative to definitely positive.

Analysis of the data was performed using the Statistical Package for Social Sciences (SPSS) program for Windows (version 16, Chicago, IL). The kappa statistical test was used to measure intra-examiner reliability. Simple descriptive statistics (percentages and frequencies) of different variables were assessed. Pearson’s Chi-square test was used to determine the association between the groups and variables. The clinical parameters (OHI, PI, GI, and caries scoring) were analyzed using Student’s t-test. A *P* < 0.05 was considered to be significant. For the purpose of data analysis, the levels of the indices of gingival status were given numerical values: poor = 1, fair = 2, and good = 3 for OHI, and mild = 1, moderate = 2, and severe = 3 for PI and GI.

## Results

Intra-examiner reliability for the OHI, PI, GI, and caries scoring was measured using the kappa test and gave results ranging between 0.7 and 1.0. Oral examination was conducted for 79 visually impaired children and 83 sighted children. The age of the children ranged from 6 to 12 years of age with an average age of 9.81 ± 2.0 years for the visually impaired group and 9.21 ± 1.8 years for the control group.

**Fig. 1:**
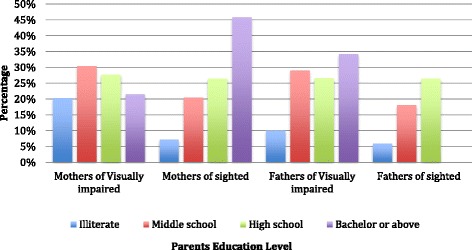
Distribution of the study groups according to parents’ education

Out of the 162 questionnaires distributed, the same number was received back, giving a response rate of 100%. Regarding the mothers’ education, 20% of the mothers in the visually impaired group were illiterate compared with 7% of the mothers of sighted children, and this difference was found to be statistically significant (*P* = 0.003). No statistically significant differences were found regarding the fathers’ education between the two groups (Fig. 1).

The general health of the children of both groups was reported as good. Nearly 22% of the visually impaired group had systemic diseases compared with only 4.8% of the sighted group (*P* = 0.002). Psychological problems, epilepsy and diabetes were the most common conditions among the visually impaired children (Table 1).

**Table 1:** Distribution of the study groups according to their reported medical health status and medical conditions

Medical condition	Visually impaired N (%)	Sighted N (%)	*P*-value*
Medical health status			
Healthy	62 (78.5)	79 (95.2)	0.002
Diseased	17 (21.5)	4 (4.8)	
Chronic medical conditions			
Heart problems	2 (2.5%)	1 (1.1%)	
Liver disease	0 (0%)	1 (1.2%)	
Kidney	0(0%)	0 (0%)	
Asthma	2(2.5%)	1 (1.2%)	
Diabetes	3 (3.8%)	1 (1.2%)	
Epilepsy	3(3.8%)	1(1.2%)	
Psychological problems	4 (5.1%)	1(1.2%)	
Speech difficulty	1 (1.3%)	0 (0%)	
Others	2(2.5%)	0(0%)	
Total	17	6	

*P-value for Pearson’s Chi-square test

Approximately 71% of the sighted children had been to the dentist compared with 54.5% of the visually impaired children (*P* = 0.028). With regard to oral hygiene practices, 90.4% of the sighted children stated that they brushed their teeth, compared with only 78.5% of the visually impaired children, and this difference was found to be statistically significant (*P* = 0.043).

Regarding the OHI, statistically significant differences (*P* = 0.001) were found between the two groups. Nearly half of the sighted children had good oral hygiene compared with only 22.8% of the visually impaired group. The visually impaired children had mostly fair oral hygiene (58.2%). Both the visually impaired and sighted children showed varying degrees of plaque accumulation ranging from mild to moderate. The results showed statistically significant differences in plaque score between the two groups (*P* < 0.0001), as moderate accumulations of plaque were found more frequently among the visually impaired group. Most children in both groups exhibited mild gingivitis; however, a higher percentage of the visually impaired children showed moderate gingivitis (26.5%) compared with only 6.9% of the sighted group (Table 2).

**Table 2:** Comparison of mean scores of oral hygiene, plaque and gingival status among visually impaired and sighted children

Index	Visually impaired (*n* = 79)	Sighted (*n* = 83)	*P*-value*
	Mean (SD)	Mean (SD)	
OHI	2.04(0.64)	2.41(0.64)	0.001
PI	1.72 (0.59)	1.32 (0.52)	< 0.0001
GI	1.35 (0.56)	1.06 (0.25)	0.001

*OHI* Oral hygiene index, *PI* Plaque index, *GI* Gingival index, *SD* Standard deviation

*P-value for t-test

No statistically significant differences were found between the two groups in terms of mean dmfs values (*P* = 0.257). However, statistically significant differences (*P* = 0.039) were found with regard to DMFS score between the two groups, with a higher mean DMFS score (5.16 ± 8.1) among the visually impaired children compared with the sighted group (3.10 ± 3.7). Regarding the DMFT and dmft, no significant differences were found between the two groups, although high mean values were noted for both groups (Table 3).

**Table 3:** Comparison of caries status among visually impaired and sighted children

Index	Visually Impaired	Sighted	*P*-value*
	Mean (SD)	Mean (SD)	
DMFS	5.16 (8.1)	3.10 (3.7)	0.03
dmfs	9.77 (11.1)	11.90 (12.6)	0.25
DMFT	2.13 (2.63)	2.77 (3.24)	0.53
dmft	4.22 (3.93)	3.91 (3.41)	0.54

SD-Standard deviation DMFS-Decayed, Missing and Filled surface for permanent teeth dmfs-decayed, missing and filled surface for primary teeth DMFT-Decayed, Missing and Filled teeth for permanent teeth dmft-decayed, missing and filled teeth for primary teeth

*P-value for t-test

Statistically significant differences were found between the two groups in terms of having received information about oral health (*P* = 0.046). The highest percentage of both groups received their information from their parents (Table 4).The majority of the examined children showed positive behavior. Although a statistically significant difference (*P* = 0.001) was found between the two groups, as 11.4% of the visually impaired children displayed negative behavior compared with 0% of the sighted group.

**Table 4:** Child receiving information about oral health and the source of this information

Group	Visually impaired (*n* = 79) Yes N (%)	Sighted (*n* = 83) Yes N (%)	*P*-value*
Did the child receive information about oral health?	60 (75.9)	73 (88)	0.046
If yes:			
Source of information: ^a^			
- Parents	38 (48.1)	62 (74.7)	0.001
- Siblings	3 (3.8)	6 (7.2)	0.341
- Teachers	32 (40.5)	14 (16.9)	0.001
- Dentists	31 (39.2)	49 (59.0)	0.012
- Media	0 (0.0)	3 (3.6)	0.088

*P-value for Pearson’s Chi-square test

^a^Multiple responses allowed

## Discussion

This comparative study provided information on the medical and dental health status, and the oral health knowledge of visually impaired and sighted female children aged 6–12 years living in Riyadh, Saudi Arabia. A higher percentage of the mothers of the visually impaired children were illiterate. Neglecting themselves and their education might result from their children occupying so much of their time. The high percentage of illiterate mothers among the visually impaired group may have contributed to the lower percentages of participants with good OHI scores, which in turn caused higher levels of GI and DMFS within the stated group. This is in accordance with a study that found that children’s oral health status is often related to social dimensions such as parental income and education [22].

Nearly a quarter of the visually impaired children had reported medical problems, the incidence of which was lower among the control group. This might be related to visually impaired children’s regular visits to the doctor for their vision problems, which may aid in the early diagnosis of any other medical conditions. The distribution of psychological problems was higher among the visually impaired group, as vision loss may be associated with depression, anxiety, and negative psychosocial consequences that affect everyday life. This is consistent with the findings of a previous study [11]. This may arise because of feelings of inadequacy, and facing difficulties in social interactions and making contacts [11]. Therefore, social support is important and needed from both family members and communities, and may help ameliorate the consequences of vision loss. Medical condition such as diabetes, epilepsy and physiological problems have been proven to affect oral health status and are common factors in the development of periodontal disease [23, 24]. These conditions might have increased the GI scores among the visually impaired group.

This study showed that dental visits by the visually impaired group were infrequent compared with in the sighted children. Although the frequency of dental visits might be related to the guardian’s compliance, the percentage of children regularly visiting a dentist was significantly lower than in the control group. This might be because the parents of visually impaired children are preoccupied with managing more pressing disability-related issues and consequently neglect dental care.

In general, most of the children had acceptable oral hygiene but a higher proportion of the sighted children had good oral hygiene compared with the visually impaired children. This result was consistent with earlier studies [4, 13]. This might in part be due to the sensory impairment, which makes maintaining good oral hygiene more difficult for the visually impaired group [1, 2]. However, in a study done in 2013, visually impaired children living in an institutional arrangement showed more consistent oral hygiene compared with sighted children because their caregivers enforced a mandatory oral hygiene routine [1]. This shows that visually impaired children area as capable of maintaining adequate oral hygiene as sighted children, as long as they are provided with adequate and consistent supervision.

The visually impaired children showed higher scores for plaque accumulation, consistent with Bimstein et al. (2013) [3]. This high plaque score led to more gingival inflammation, which was also significantly different between the two groups. More than one quarter of the visually impaired children showed moderate gingivitis; therefore, prevention programs directed toward the visually impaired children are essential. These programs can achieve promising results when proper communication methods are used with the patient as well as their caregivers. There are several methods that can help to increase the efficiency of implementing such programs in such groups. These include: avoiding wearing masks while speaking to the visually impaired; lessening background sounds; avoiding the use of visual aids; increasing the involvement of the child’s caregivers (parents and/or teachers); and regular oral health checkups to evaluate how well the dental hygiene instructions were received and how well they are being implemented [3].

In this study, the visually impaired children experienced higher mean DMFS scores when compared with their sighted peers. This finding is comparable to a study conducted in Turkey [5]. However, in primary dentition, the sighted children had higher dmfs values. These results are consistent with a previous study conducted in Turkey [5]. High mean DMFT and dmft values were found among both groups, which supports the high caries prevalence found in a previous study among the general child population of Riyadh [25].

The visually impaired children had received less information regarding oral health compared with the sighted group, which was consistent with the results of Chang and Shih (2004) [9]. This could be due to the difficulty visually impaired children may have in visualizing their oral health, and in understanding its importance. Parents’ role in informing the children regarding their oral health was more pronounced in the control group in comparison with the visually impaired children. This may be due to the lack of dental educational programs available for dentists that are directed toward groups with disabilities, their families, and caregivers. Therefore, special educational programs with a greater emphasis on oral health and oral hygiene practices for visually impaired children and their parents should be provided. Teachers were reported as a source of information by more than one third of the visually impaired group. This is contrary to a study by Bekiroglua et al. [26], which found that only 6.5% of visually impaired children were informed about oral health by their teachers. The lack of media participation in providing knowledge about oral health to both groups was also noticeable. This finding is in keeping with those of a study in Turkey, which showed that only 1.1% of visually impaired children reported having used the media as a source of information [26]. Further attention is required from the Ministry of Culture and Information to raise the priority of oral health and advocate practicing oral habits and dental hygiene, thus playing a more active role in educating society regarding their oral and general health.

Lack of cooperation was noted among the visually impaired children, compared with completely positive behavior in their counterparts. This could be related to their inability to see, which can make it difficult for them to relax and cooperate. Knowing the best way to communicate with visually impaired children and familiarizing them with the dental setting is the responsibility of the dental professional, and could help in improving the experience for these children [5].

More attention from the responsible authorities should be directed toward these groups of children, and oral health educational, preventive and curative programs need to be developed to accommodate their needs.

One of the limitations in this study was the inability to include males in the study sample, as well as the inability of female dentists to enter male schools and perform examinations because of social segregation. There was also difficulty in dealing with the institutions caring for the visually impaired children, and their cooperation was limited. Further studies should be conducted comparing both males and females and including other age groups, as well as in populations from other areas of the kingdom.

## Conclusions

Visually impaired children in Riyadh, Saudi Arabia had more reported medical problems compared with their sighted peers. Poor OHI scores, higher PI scores, and higher GI scores were observed among the visually impaired children compared with the sighted group. They also visited the dentist and practiced tooth brushing less often. Furthermore, the sighted group received more information regarding oral health than the visually impaired children.

## Abbreviations

ANOVA: One-way analysis of variance
DMFT/ DMFS: Decayed, Missing and Filled Teeth/Surfaces for permanent teeth
dmft/ dmfs: decayed, missing and filled teeth/surfaces for primary teeth
GI: Gingival index
OHI: Oral hygiene index
PI: Plaque index
SPSS: Statistical Package for Social Sciences
WHO: World Health Organization
